# Lean mass as a risk factor for intensive care unit admission: an observational study

**DOI:** 10.1186/s13054-021-03788-y

**Published:** 2021-10-18

**Authors:** Matthew Thackeray, Mohammadreza Mohebbi, Neil Orford, Mark A. Kotowicz, Julie A. Pasco

**Affiliations:** 1 IMPACT (Institute of Mental and Physical Health and Clinical Translation), Deakin University, Geelong, Australia; 2 Barwon Health, Geelong, Australia; 3 Faculty of Health, Biostatistics Unit, Deakin University, Geelong, Australia; 4 Australian and New Zealand Intensive Care Research Centre (ANZIC-RC), Department of Epidemiology and Preventive Medicine (DEPM), Monash University, Melbourne, Australia; 5 Department of Medicine-Western Health, The University of Melbourne, St Albans, Australia; 6 Department of Epidemiology and Preventive Medicine (DEPM), Monash University, Melbourne, Australia

**Keywords:** Intensive care unit, Muscle mass, Lean mass, DXA, Sarcopenia, Outcomes

## Abstract

**Purpose:**

Intensive care unit (ICU) survivors have reduced physical function likely due to skeletal muscle wasting and weakness acquired during critical illness. However, the contribution of pre-morbid muscle mass has not been elucidated. We aimed to examine the association between pre-ICU muscle mass and ICU admission risk. Secondary outcomes include the relationship between muscle mass and ICU outcomes.

**Methods:**

ICU admissions between June 1, 1998, and February 1, 2019, were identified among participants of Geelong Osteoporosis Study (GOS), a population-based cohort study. Cox proportional hazard regression models estimated hazard ratios (HR) for ICU admission across T-score strata and continuous values of DXA-derived lean mass measures of skeletal mass index (SMI, lean mass/body mass %) and appendicular lean mass corrected for height (ALM/h^2^, kg/m^2^). Multivariable regression was used to determine the relationship between lean mass and ICU outcomes.

**Results:**

One hundred and eighty-six of 3126 participants enrolled in GOS were admitted to the ICU during the follow-up period. In adjusted models, lean mass was not predictive of ICU admission (SMI: HR 0.99 95%CI 0.97–1.01, *p* = 0.32; ALM/h^2^: HR 1.11 95%CI 0.94–1.31, *p* = 0.23), while greater appendicular lean mass was related to reduced 28-day mortality (ALM/h^2^ adjOR: 0.25, 95%CI 0.10–0.63, *p* = 0.003, SMI adjOR: 0.91, 95%CI 0.82–1.02, *p* = 0.09).

**Conclusion:**

Lean mass was not associated with ICU admission in this population-based cohort study; however, greater appendicular lean mass was associated with reduced mortality. This suggests pre-ICU muscle status may not predict development of critical illness but is associated with better survival after critical illness occurs.

**Supplementary Information:**

The online version contains supplementary material available at 10.1186/s13054-021-03788-y.

## Introduction

Survivors of Intensive Care Unit (ICU) admission are identified as having reduced physical function in the years following their critical illness [1–5]. This is proposed to be part of the long-term sequelae of acute skeletal muscle wasting and neuromuscular weakness sustained during critical illness due to prolonged immobilisation, systemic inflammation and bioenergetic failure [6–11].

While older and more co-morbid individuals are at increased risk of physical impairment and/or decline after ICU admission [12–15], it is not clear whether the long-term physical limitations experienced by ICU survivors are completely or partially attributable to the impairments acquired during their time in the ICU, or to a continuation of pre-admission functional impairment caused by chronic disease or general frailty [12, 16–18].

The challenge in determining the relationship between pre-admission physical status and long-term outcomes is the ascertainment of accurate pre-ICU patient status, given the mostly random nature of ICU admission. The use of population-based longitudinal cohort study data has provided opportunities to examine unbiased pre-post ICU objective measures, with existing studies providing conflicting evidence for newly acquired post-ICU physical limitations versus a continuation of functional trajectory before and after critical illness [19, 20].

There is an established relationship between muscle mass and physical performance and function [21–23]. Acute loss of muscle mass in the ICU has been demonstrated extensively in the literature, but it is not known if ICU populations arrive to the ICU with pre-existing low muscle mass. Existing data examining pre-admission muscle status in ICU patients are limited to specific chronic disease populations [24, 25]. Quantification of pre-ICU muscle mass in the broader ICU population and its potential contribution to post-ICU functional impairment is required.

This study aims to examine the association between pre-ICU lean mass and admission to the ICU using data from an existing population-based prospective study. We hypothesised that participants with lower pre-ICU lean mass are at an increased risk of admission to the ICU. We also aimed to examine the relationship between pre-admission lean mass and ICU outcomes.

## Methods

### Study design

A retrospective observational study was performed including all participants from the Geelong Osteoporosis Study (GOS). Participants from the GOS with an admission to ICU were identified by data linkage to determine risk of admission based on lean mass measures. Ethics approvals were obtained from the Barwon Health Human Ethics Research Committee. All participants provided informed, written consent and consented to use of hospital data at the time of assessment.

### Study population

The GOS is a population-based cohort study established to investigate the epidemiology of osteoporosis in Australia [26]. It comprises adult participants randomly selected, using the electoral roll as a sampling frame, from the Barwon Statistical Division in Victoria, south-eastern Australia, where voting is compulsory. This region has population characteristics comparable with the national population. Female participants were first enrolled in 1993, with enrolment of male participants commencing in 2001. Participants undergo an array of measures including regional and whole-body dual energy X-ray absorptiometry (DXA) at baseline and regular follow-up intervals (2, 4, 6, 8, 10 and 15 yr for women and 5 and 15 yr for men).

The University Hospital Geelong (UHG) ICU is a 24-bed tertiary regional mixed medical, surgical, and cardiothoracic ICU situated in Geelong, Australia. It serves a catchment of approximately 500,000 people and is the only tertiary ICU in the Barwon Statistical Division. Data were available for ICU admissions from 1 of June 1998 to 1 of February 2019.

Participants from the GOS with an admission to UHG ICU were identified via data linkage of the GOS and UHG ICU databases. Participants with ICU admission without available pre-admission DXA imaging were excluded from the analysis. For participants with multiple ICU admissions, data from their first admission were used.

### Data collection

Demographic data for GOS participants included age, sex, date of birth and if applicable, date of death from the National Death Index (NDI). Data collected at each study visit included: the date of visit, height measured to the nearest 0.1 cm using a wall-mounted Harpenden stadiometer, weight measured to the nearest 0.1 kg and body mass index (BMI) was calculated as weight/height^2^ (kg/m^2^). DXA whole body scans were also performed at each study visit providing measures of whole-body lean mass, whole body fat mass, whole body bone mass and lean mass of each limb. Initial scanning of both men and women was via a DPX-L scanner (Lunar, Madison, WI, USA) until it was replaced with a Prodigy Pro (Lunar) during baseline assessment of men and after 10-year follow-up for women. The sum of lean mass of the four limbs was used to calculate appendicular lean mass (ALM). Skeletal Mass Index (SMI) and relative Appendicular Lean Mass (ALM/h^2^, kg/m^2^) are the two measures of lean mass used in this study as surrogate measures of muscle mass. SMI represents whole body lean mass expressed as a percentage of total body mass, and ALM/h^2^ was calculated as appendicular lean mass corrected for height. An ICU electronic database provided information on ICU and hospital admission date, ICU, and hospital length of stay (LOS), ICU and hospital survival, admission category, APACHE II score, co-morbidities, and duration of mechanical ventilation. To determine if the GOS-ICU sample was representative of the general UHG ICU population, these data were also collected for all admission to UHG ICU during the study period.

### Outcomes

The primary outcome was relative risk of ICU admission based on measures of lean mass expressed as continuous variables or T-score strata. For the ICU-GOS participants, secondary outcomes included hospital and ICU survival, hospital and ICU length of stay, and duration of mechanical ventilation.

### Statistical analysis

Characteristics of participants were described by mean (± SD) or median (IQR) or relative frequencies (%) stratified by ICU admission, ICU, or in-hospital mortality status. Participant characteristics were compared using *t*-test or Mann–Whitney *U* test for continuous data and *χ*^2^ test (or Fisher’s exact test) for categorical data. ICU admission rates (per 1000 person-year) based on SMI and ALM/h^2^ T-score strata were calculated. Cox proportional hazard regression models were used to estimate hazard ratios (HR) and 95% CIs for ICU admission status across T-score strata of SMI or ALM/h^2^. T scores for SMI were calculated using the full dataset to calculate the young adult (20–39 yr) mean (men = 74.10 ± 7.37 [*n* = 356], women = 59.62 ± 8.99 [*n* = 315]), and published T-score values for ALM/h^2^ were used [27]. Age and sex adjusted HRs were estimated from multivariable Cox proportional hazard regressions. The proportional hazard assumption was evaluated graphically by log(-log(survival)) plots, and Kaplan–Meier survival curves and model-based survival plots after adjusting for sex and gender for T-score strata of SMI and ALM/h^2^ were illustrated.

Multivariable logistics regression was used to estimate odds ratio (OR) and 95% CIs for ICU, hospital, and 28-day mortality across continuous scores and proportions status across T-score strata of SMI and ALM/h^2^. The multivariable logistic models were adjusted for age, sex, APACHE III score, admission category, co-morbidities, and smoking status, which were determined a priori based on known risk factors for mortality. Due to potential improvement in mortality rates during the study period, the analysis was repeated with the addition of ICU admission year in the form of five-yearly strata.

Multivariable linear regression analysis was used to model the association between SMI or ALM/h^2^ with ICU, hospital length of stay (LOS) or length of mechanical ventilation. Due to skewness, ICU and hospital LOS were log-transformed. The covariates included in each model were the same as previously described for logistic models. A two-sided alpha level of 0.05 was used to determine statistical significance. Stata 16 (StataCorp. 2019. Stata Statistical Software: Release 16. College Station, TX: StataCorp LLC) was used for statistical analysis.

## Results

### Participants

**Fig. 1:**
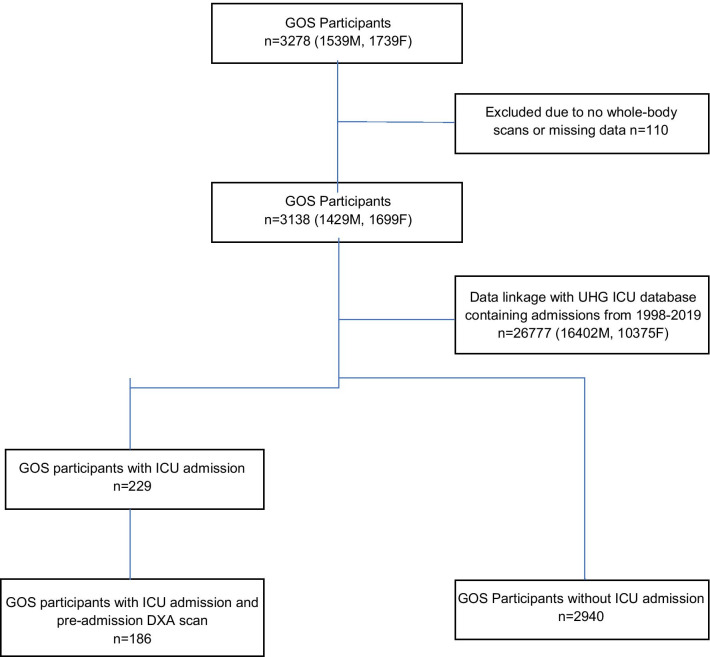
Flowchart for included participants

Between June 1998 and February 2019, across 48.155 person-years, data linkage identified 186 GOS participants with admission to UHG ICU and at least one pre-admission DXA scan, with multiple admissions among 31 participants (Fig. 1).

At enrolment to GOS, baseline comparison of the ICU and non-ICU GOS participants revealed the ICU-GOS group had a higher proportion of men, were older, and had a higher BMI (Table 1). Due to its nonlinear relationship with ICU admission, age was expressed as a categorical variable with four strata (< 40, 40–59, 60–79 and ≥ 80 yr). Most participants admitted to the ICU were over the age of 60 years, with two and 21 participants admitted to the ICU from the < 40 yr and 40–60 yr age strata, respectively. A high proportion of ICU-GOS participants were admitted following surgery (38.2% general surgery, 35.5% cardiothoracic surgery), and cardiovascular co-morbidities were the most common. More than half (58.1%) of the ICU–GOS participants were mechanically ventilated, for a median duration of 12 h [IQR 8, 22], with ICU mortality 7.1%, and hospital mortality 13.1%. Compared to all admissions to UHG ICU during the study period, there were no differences in proportion of males, ICU- or hospital LOS, duration of mechanical ventilation, or ICU mortality rate for the GOS-ICU participants. However, our sample was older at ICU admission (median 75 yr [IQR 68, 81] vs 66 yr [IQR 54, 75], *p* < 0.001), had a higher APACHE III score (median 55 [IQR 43, 66] vs 51 [39, 65], *p* = 0.03) and contained a higher proportion of surgical patients (73.4 vs 57.8%, *p* < 0.001).

**Table 1:** Baseline characteristics of participants included in analysis

	ICU GOS	Non-ICU GOS	*P* value
Number	186	2940	–
Male/Female	115/71	1312/1628	< 0.001
Age at GOS enrolment (yr)	66.6 [59.8, 73.6]	50.5 [33.4, 70.3]	< 0.001
< 40	9 (4.8)	1027 (35.0)	
40–59	39 (21.0)	819 (27.9)	
60–79	112 (60.2)	731 (24.9)	
> 79	26 (14.0)	362 (12.3)	
Height at first scan (cm)	167.3 (± 9.4)	167.4 (± 10.2)	0.87
Weight at first scan (kg)	79.3 (± 16.8)	74.7 (± 16.1)	< 0.001
BMI at first scan (kg/m^2^)	28.4 (± 4.8)	26.6 (± 4.8)	< 0.001
Lean mass at first scan			
Total (kg)	50.4 (± 11.2)	47.4 (± 11.5)	< 0.001
Percentage (%)	64.2 (± 9.3)	64.2 (± 9.8)	0.98
Fat mass at first scan			
Total (kg)	25.4 (± 9.0)	23.7 (± 9.7)	0.03
Percentage (%)	32.2 (± 9.7)	31.6 (± 10.1)	0.43
ALM/h^2^ at first scan (kg/m^2^)	7.82 (± 1.26)	7.48 (± 1.33)	< 0.001
Age at ICU admission (yr)	75 [68, 81]	–	–
Admit category			
Medical	49 (26.3)	–	–
Surgical	71 (38.2)	–	–
Cardiothoracic surgery	66 (35.5)	–	–
Co-morbidities*			
Respiratory	68 (36.6)	–	–
Cardiovascular	118 (63.4)	–	–
Renal	26 (14.0)	–	–
Diabetes	42 (22.6)	–	–
Smoker	88 (47.3)	–	–
ICU LOS (days)	1.67 [0.94, 2.31]	–	–
Hospital LOS (days)	9.65 [6.09, 14.37]	–	–
APACHE III score	55 [44, 66]	–	–
Mechanically ventilated	108 (58.1)	–	–
Duration of ventilation (h)	12 [8, 21.5]		
Died in ICU	13 (7.1)	–	–
Died in hospital	24 (13.1)	–	–
28-day mortality	21 (11.3)		

Results are presented as median [interquartile range], mean (± standard deviation), or percentage (%)

GOS Geelong Osteoporosis Study; yr. years; cm centimetre; kg kilogram; BMI body mass index; ALM/h^2^ relative appendicular lean mass (kg/m^2^); ICU intensive care unit; LOS length of stay; Hr hours; APACHE III acute physiology, age, and chronic health evaluation system III

*At time of ICU admission

### Muscle mass as a risk factor for ICU admission

**Fig. 2:**
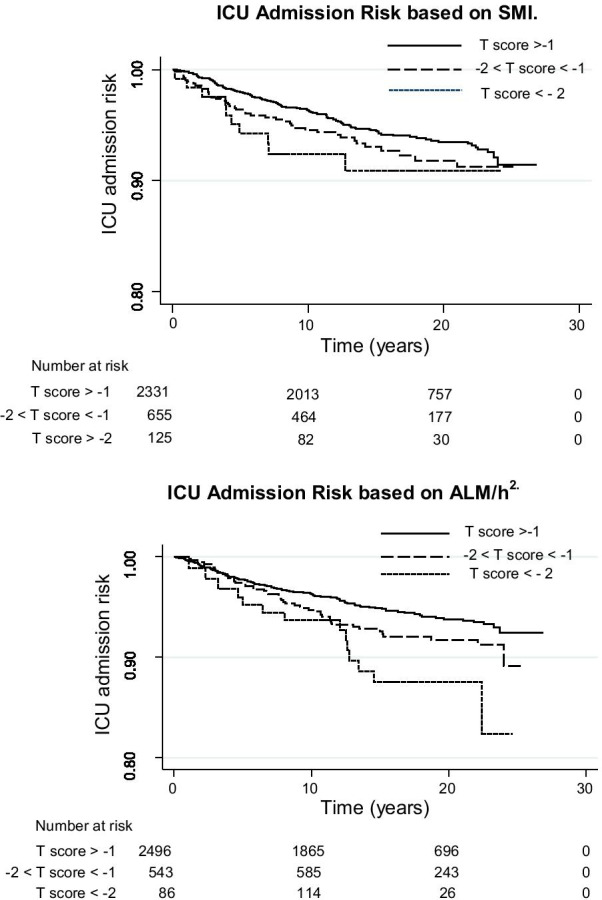
Kaplan–Meier curves for ICU admission risk. SMI: Skeletal mass index; ALM/h^2^: relative appendicular lean mass

Overall rate for ICU admission was 3.86 (95%CI 3.35–4.46) per 1000-person-years, with the rate for men more than double that of women (6.47; 95%CI 5.39–7.76 vs. 2.34; 95%CI 1.85–2.95). Overall, ICU admission rate increased with decreasing T-score strata for both ALM/h^2^ and SMI (Table 2). Kaplan–Meier curves for ICU admission risk across T score strata are presented in Fig. 2. When separated into sex, the pattern of progressive ICU admission risk with decreasing lean mass remained present for both males and females; however, differences between T scores were not significant for females due to lower numbers in each stratum.

**Table 2:** ICU admission rates (per 1000 person-year) based on percentage lean mass (SMI) and appendicular lean mass corrected for height^2^ (ALM/h^2^)

Overall	N	Rate	SMI	N	Rate	ALM/h^2^	N	Rate
Total	186	3.86 (3.35–4.46)	T > − 1	123	3.43 (2.88–4.10)	T > − 1	133	3.58 (3.02–4.24)
			− 2 < T ≤ − 1	49	4.67 (3.53–6.18)	− 2 < T ≤ − 1	43	4.67 (3.46–6.29)
			T ≤ − 2	14	7.65 (4.53–12.91)	T ≤ − 2	10	6.06 (3.26–11.26)
Males	115	6.47 (5.39–7.76	T > − 1	73	5.87 (4.67–7.39)	T > − 1	79	5.78 (4.63–7.20)
			− 2 < T ≤ − 1	32	7.13 (5.04–10.08)	− 2 < T ≤ − 1	29	8.41 (5.85–12.11)
			T ≤ − 2	10	11.50 (6.19–21.37)	T ≤ − 2	7	11.64 (5.55–24.41)
Females	71	2.34 (1.85–2.95)	T > − 1	50	2.14 (1.62–2.82)	T > − 1	54	2.30 (1.76–3.00)
			− 2 < T ≤ − 1	17	2.84 (1.76–4.56)	− 2 < T ≤ − 1	14	2.43 (1.44–4.10)
			T ≤ − 2	4	4.16 (1.56–11.09)	T ≤ − 2	3	2.86 (0.92–8.87)

SMI skeletal mass index; ALM/h^2^ relative appendicular lean mass

Person-years is a form of person-time measurement, it is summation of the actual time-at-risk that all participants contributed to the study. It is the sum of the time the participants spent in the study during the follow-up period (prior to ICU admission, death, or end of study period)

In Cox regression analysis, the unadjusted hazard ratio for ICU admission increased with decreasing T-score of lean mass but was only significant for SMI and not ALM/h^2^. After adjustment for age and sex, neither lean mass measure was a significant predictor of ICU admission risk (Table 3). Expressed as continuous variables, age- and sex-adjusted SMI or ALM/h^2^ were not predictors of ICU admission rate (SMI: HR 0.989 [95%CI 0.967, 1.010, *p* = 0.315], ALM/h^2^: HR 1.108 [95%CI 0.936, 1.311, *p* = 0.233). There were no significant interactions detected between age and sex in analysis of lean mass as either categorical or continuous variables.

**Table 3:** Comparison of ICU admission risk using T-scores of SMI and ALM/h^2^: results of unadjusted and age and sex adjusted Cox proportional hazard models

	T score	Unadjusted HR (95%CI)	SE	*P* value	AdjHR (95%CI)	SE	*P* value
SMI	T > − 1	1.00	–	–	1.00	–	–
	− 2 < T ≤ − 1	1.40 (1.01–1.95)	0.24	0.05	1.00 (0.71–1.40)	0.17	0.99
	T ≤ − 2	2.26 (1.30–3.93)	0.64	< 0.01	1.51 (0.86–2.64)	0.43	0.15
ALM/h^2^	T > − 1	1.00	–	–	1.00	–	–
	− 2 < T ≤ − 1	1.29 (0.91–1.81)	0.23	0.15	0.95 (0.67–1.34)	0.17	0.75
	T ≤ − 2	1.66 (0.87–3.16)	0.55	0.12	0.95 (0.49–1.83)	0.32	0.88

SMI skeletal mass index; ALM/h^2^ relative appendicular lean mass; HR hazard ratio; AdjHR adjusted hazard ratio, CI confidence interval; SE standard error

Post hoc power calculations were performed based on median split samples. Type I error was set at 0.05, and detectable effect size (i.e. RR) was estimated under 80% statistical power. With a total sample size of 3126 and 48,166 total person-years exposure time, an ICU admission event rate ratio of 1.4 or greater is detectable. So, the study had statistical power to detect moderate effect sizes and potentially underpowered for very small effect sizes.

### Pre-ICU muscle mass and its relationship to ICU outcomes

Of the 186 ICU-GOS participants, three were transferred to other hospitals leaving 183 participants with data on ICU and hospital outcomes. Participants who died in ICU or hospital were older, more likely to have medical ICU admission, had a higher APACHE III score, and lower ALM/h^2^. Those who did not survive hospital also had higher proportions with smoking history and respiratory or cardiovascular co-morbidity (Additional file 1: Table S1).

When expressed as a continuous variable and after adjustment for sex, age, ICU admission category, co-morbidities, APACHE III score and time from DXA scan to ICU, increasing SMI was associated with a reduction in the odds for ICU death (adjOR: 0.78: 95%CI 0.63–0.98; *p* = 0.029), whereas ALM/h^2^ was not (adjOR 0.68: 95%CI 0.25–1.87; *p* = 0.46). In contrast, ALM/h^2^ (adjOR 0.40: 95%CI 0.18 to 0.90; *p* = 0.027) but not SMI (adjOR 0.92: 95%CI 0.84–1.01; *p* = 0.08) was associated with reduced odds of in-hospital death. Increased relative appendicular lean mass was associated with reduced 28-day mortality, while percentage lean mass was not (ALM/h^2^ adjOR: 0.25, 95%CI 0.10–0.63, *p* = 0.003, SMI adjOR: 0.91, 95%CI 0.82–1.02, *p* = 0.09). Admission year was not associated with ICU, hospital, or 28-day mortality and did not alter the results for lean mass. Pearson *χ*^2^ test was performed to compare the distribution of ICU mortality across T score strata of lean mass. There was a significant relationship between ALM/h^2^ and ICU mortality (*χ*^2^ = 9.38, *p* 0.009) and hospital mortality (*χ*^2^ = 23.54, *p* < 0.001) but not SMI (ICU mortality *χ*^2^ = 1.47, *p* = 0.48; hospital mortality *χ*^2^ = 3.19, *p* = 0.2). However, low numbers (< 5) in expected frequency counts for several T score strata limit the reliability of these results. In multivariable linear regression analysis of 186 GOS-ICU participants, neither measure of pre-admission lean mass was a predictor of ICU LOS (SMI Beta 1.01, 95%CI 0.99–1.02, *p* = 0.55; ALM/h^2^ Beta 1.14, 95%CI 0.98–1.32, *p* = 0.08) or hospital LOS (SMI Beta 1.00, 95%CI 0.98–1.02, *p* = 0.82; ALM/h^2^ Beta 1.02, 95%CI 0.88–1.18, *p* = 0.82) when expressed as a continuous variable. When lean mass values were expressed as a categorical variable in the form of T score strata, results remained unchanged. In 108 ICU patients who received mechanical ventilation, lean mass was not related to duration (SMI Beta 1.02, 95%CI 0.99–1.06, *p* = 0.20; ALM/h^2^ Beta 1.16, 95%CI 0.93–1.45, *p* = 0.19).

## Discussion

In this study, we aimed to determine the association between muscle mass and admission to the ICU, using DXA-derived measures of pre-ICU lean mass, in participants from a population-based cohort study. Lean mass expressed as percentage lean mass or relative appendicular lean mass was not a significant predictor of ICU admission, indicating that we did not detect a difference in lean mass between ICU and non-ICU participants prior to ICU admission. However, greater pre-ICU percentage lean mass (SMI) was associated with reduced ICU mortality, suggesting a relationship between relative lean mass rather than absolute lean mass. Interestingly appendicular lean mass but not percentage lean mass was associated with in-hospital mortality. Finally, our observation of a twofold increase in ICU admission rate among men has not previously been reported in the Australian setting and could represent a previously described sex-bias [28, 29].

This is the first known study to use population-based data to examine pre-ICU muscle mass with previous research existing only in specific subgroups without comparison with population norms. Accelerated muscle loss (> 2% per year) measured via computed tomography (CT) at the third lumbar spine was reported in 68% of liver cirrhosis patients admitted to the ICU [25] while in the liver and lung transplant populations lower pre-operative muscle mass is common (32–35%) [24, 30, 31]. These are very specific ICU admission sub-groups where low muscle mass is likely to be a surrogate marker of more severe chronic disease. Low muscle mass estimated via bioelectrical impedance analysis was less common (8.3%) in elective cardiac surgery patients [32], in keeping with the results of this study, which contains a high proportion of cardiothoracic surgery patients.

Our results show that lean mass does not predict which community-based adults will require ICU admission and indicate that these patients arrive at ICU with normal muscle mass. However, the ICU admissions from the cohort in this study comprised predominately surgical patients who underwent mechanical ventilation for relatively short periods of time with short ICU length of stay. Surgical patients have been shown to have lower risk of new functional impairment at one year following discharge (OR 0.3; 95%CI 0.2–0.44) [4] compared to medical ICU patients while known risk factors for post-ICU physical impairment from existing literature include age, history of anxiety, co-morbidities, sepsis, duration of mechanical ventilation and ICU length of stay [3, 4, 33, 34]. This cohort was older and had more co-morbidity, but other risk factors were absent, indicating their risk of functional impairment may be lower than other ICU patients admitted with sepsis, respiratory failure, acute respiratory distress syndrome or trauma, where much of the existing research has been conducted [2, 33, 35–37]. The GOS is also an ambulant population-based sample which may not be representative of the entire ICU population regarding pre-ICU muscle mass.

Muscle mass and quality measured within 48 h of ICU admission has been shown to be predictive of ICU outcomes including mortality [38–41]. However, acute illness is likely to have commenced days to weeks preceding ICU admission and with the early commencement of skeletal muscle wasting in critical illness [6], these measures are not representative of pre-admission muscle mass. Existing literature examining the association of true pre-admission muscle mass with ICU and hospital outcomes has produced varying results, possibly due to inconsistencies in measurement approaches and cut-offs for low muscle mass, and heterogeneity between population groups. Measurement at the L3 vertebra or psoas via clinically obtained abdominal or pelvic computed tomography are common approaches. Muscle mass is an independent predictor of hospital mortality in elderly surgical patients, is associated with duration of mechanical ventilation in lung transplant patients and increased ICU and hospital mortality in liver cirrhosis patients [24, 25, 42]. In contrast, muscle mass is not a predictor of hospital mortality in non-elderly surgical patients, and low muscle mass was not associated with mortality or ICU length of stay in cardiac surgery patients [32]. These studies used non-validated or regional measures of muscle mass and included specific ICU sub-populations.

Our study used a reliable measure of lean mass (DXA) in a general ICU population and observed a relationship between pre-ICU percentage lean mass and ICU mortality. Percentage lean mass represents body composition and has an inverse relationship with percentage fat mass. This suggests a protective effect of lean mass relative to fat mass or could be due to increased fat mass itself. In contrast, hospital and 28-day mortality were associated with relative appendicular lean mass. Muscle mass of the limbs is likely to be more closely associated with strength and function than percentage lean mass, and lower appendicular lean mass could be a surrogate for increased frailty. Frailty is defined by a lack of physiological reserve and compromised ability to cope with stressors [43], such as critical illness, and therefore may be more closely associated with overall survival. In keeping with previous research reporting muscle mass as a predictor of all-cause mortality [44], our finding supports the need to maintain muscle mass across the lifespan. Given the high proportion of surgical patients in this cohort, our findings could support a role in pre-operative rehabilitation to improve post-surgical ICU outcomes.

Muscle mass is only one determinant of physical function, and an absence of difference between ICU and non-ICU participants does not exclude a difference in physical function. Muscle quality, strength and performance are also important measures in the overall health of an older population and could potentially predict ICU admission, given that pre-admission low grip-strength and walking speed have been associated with poorer acute and long-term outcomes in the ICU population [45].

### Limitations and strengths

A strength of this study is the use of data from a population-based cohort study providing the opportunity for objective measurement of pre-admission lean mass in ICU patients who were not selected based on disease. The use of whole-body measures via DXA is a precise method for evaluating whole-body and appendicular lean mass and its use in this study is in keeping with recommendations for assessment of lean mass as a surrogate measure for skeletal muscle mass in sarcopenia research [46, 47]. Limitations of this study include the large proportion of surgical patients in the GOS participants admitted to ICU, potentially limiting the extrapolation to non-surgical ICU populations. We collected data from the sole tertiary level ICU in the region; however, it is possible that admissions to an ICU outside of the area were unaccounted for. It is not known if frailer and sarcopenic patients were not admitted to the ICU due to poor prognosis and whether this has limited our power to detect a relationship between muscle mass and ICU admission. Due to the intervals in follow-up in GOS, we are also not able to account for any changes in lean mass in the time between DXA scanning and ICU admission. Finally, with the use of longitudinal observational studies there is a risk of loss to follow-up bias.

## Conclusion

This study examined the association between lean mass and the risk for admission to the ICU. We report that DXA-derived lean mass was not identified as an independent predictor for ICU admission; however, a greater percentage lean mass was associated with lower ICU mortality. Further research examining pre-post changes in muscle mass in ICU patients and the association with functional outcomes will assist in determining the relationship of pre-morbid muscle with post-ICU impairments.

## Supplementary Information

**Additional file 1**. Baseline characteristics of GOS-ICU participants based on hospital survival.

## Data Availability

Data available upon reasonable request.

## Abbreviations

ICU: Intensive care unit
GOS: Geelong Osteoporosis Study
HR: Hazard ratio
DXA: Dual x-ray absorptiometry
SMI: Skeletal mass index
ALM: Appendicular lean mass
UHG: University Hospital Geelong
NDI: National death index
BMI: Body mass index
APACHE: Acute physiology and chronic health evaluation
OR: Odds ratio
LOS: Length of stay
CT: Computed tomography

## Code availability

Stata 16 (StataCorp. 2019. Stata Statistical Software: Release 16. College Station, TX: StataCorp LLC.) was used for statistical analysis.
