# Supplementary material for: Lean mass as a risk factor for intensive care unit admission: an observational study
Source: Crit Care. 2021 Oct 18;25:364. doi: 10.1186/s13054-021-03788-y (PMC8525013; doi:10.1186/s13054-021-03788-y)
Supplement: Supplementary file 1 — Additional file 1. Baseline characteristics of GOS-ICU participants based on hospital survival. [file 13054_2021_3788_MOESM1_ESM.docx]

**Critical Care**

**Title:** Lean mass as a risk factor for Intensive Care Unit admission: an observational study

**Authors:** Matthew Thackeray ^1,2^, Mohammadreza Mohebbi^1,3^, Neil Orford ^1,2,4^, Mark A Kotowicz ^1,2,5^, Julie A Pasco^1,2,5,6^

**Author affiliations:**

1. Deakin University, IMPACT (Institute of Mental and Physical Health and Clinical Translation), Geelong, Australia
2. Barwon Health, Geelong, Australia
3. Deakin University, Faculty of Health, Biostatistics Unit, Geelong, Australia
4. Australian and New Zealand Intensive Care Research Centre (ANZIC-RC) Department of Epidemiology and Preventive Medicine (DEPM), Monash University, Melbourne, Australia
5. Department of Medicine-Western Health, The University of Melbourne, St Albans, Australia
6. Department of Epidemiology and Preventive Medicine (DEPM), Monash University, Melbourne, Australia

**Contact:** Matthew Thackeray, [mthack@barwonhealth.org.au](mailto:mthack@barwonhealth.org.au)

**Additional file 1.**

### **Table 4**. Baseline characteristics of GOS-ICU participants based on hospital survival.

|  | Survived hospital | Died in hospital | P value |
| --- | --- | --- | --- |
| Number | 159 | 24 |  |
| Age (yr) | 72.5 (±11.5) | 81.8 (±9.0) | <0.001 |
| Male sex | 102 (64.2) | 12 (50) | 0.18 |
| Height (cm) | 167.5 (±9.8) | 163.1 (±9.3) | 0.04 |
| Weight (kg) | 80.6 (±15.7) | 75.9 (±13.4) | 0.16 |
| Admit category  Medical  Surgical  Cardiothoracic surgery | 31 (19.5)  62 (39.0)  66 (41.5) | 17 (70.8)  7 (29.2)  0 (0) | <0.001  0.35  - |
| APACHE III score | 52 [42-61] | 87 [69.5-110] | <0.001 |
| ICU LOS | 1.7 [1.0-2.1] | 1.9 [0.7-5.4] | 0.54 |
| Hospital LOS (d) | 10.1 [6.3-14.8] | 6.1 [2.0-13.6] | 0.03 |
| ICU Survival | 159 (100) | 11 (45.8) | - |
| Mechanically ventilated  Duration of mechanical ventilation (hrs) | 93 (58.5)  11 [7-19] | 15 (62.5)  46 [14-168] | <0.001 |
| Co-morbidities  Respiratory  Cardiovascular  Renal  Diabetes  Smoker | 58 (36.5)  101 (63.5)  23 (13.2)  37 (23.3)  77 (48.4) | 15 (62.5)  15 (62.5)  3 (12.5)  5 (20.8)  10 (41.7) | 0.92  0.92  0.80  0.79  0.54 |
| Time from DXA scan to ICU admission | 1211.6 [650.7-2102.9] | 1054.0 [645.5-2013.7] | 0.80 |
| SMI (%)  T score > -1  T score > -2 ≤ -1  T score ≤ -2 | 64.04 [62.55-65.54]  106 (66.7)  43 (27.0)  10 (6.3) | 59.61 [55.63-63.59]  14 (58.3)  6 (25.0)  4 (16.7) | 0.04 |
| ALM/h^2^ (kg/m^2^)  T score > -1  T score > -2 ≤ -1  T score ≤ -2 | 7.88 [7.68-8.06]  119 (74.8)  36 (22.6)  4 (2.5) | 6.96 [6.50-7.42]  10 (41.7)  8 (33.3)  6 (25.0) | <0.001 |

1. Results are presented as median [interquartile range], mean (+­ standard deviation), or percentage (%)

2. Abbreviations: cm centimetres, kg kilograms, APACHE III acute physiology, age, and chronic health evaluation system III, hr hours, DXA dual x-ray absorptiometry, ICU intensive care unit, SMI skeletal mass index, ALM/h^2^ appendicular lean mass/height2 (kg/m^2^)
